# Influence network linkages across implementation strategy conditions in a randomized controlled trial of two strategies for scaling up evidence-based practices in public youth-serving systems

**DOI:** 10.1186/1748-5908-8-133

**Published:** 2013-11-14

**Authors:** Lawrence A Palinkas, Ian W Holloway, Eric Rice, C Hendricks Brown, Thomas W Valente, Patricia Chamberlain

**Affiliations:** 1 School of Social Work, University of Southern California, Los Angeles, CA, USA; 2 Department of Social Welfare, Luskin School of Public Affairs, University of California, Los Angeles, Los Angeles, CA, USA; 3 Department of Psychiatry and Behavioral Sciences, Feinberg School of Medicine, Northwestern University, Chicago, IL, USA; 4 Department of Preventive Medicine, Keck School of Medicine, University of Southern California, Los Angeles, CA, USA; 5 Oregon Social Learning Center, Eugene, OR, USA

**Keywords:** Implementation, Randomized controlled trial, Design, Social networks, Evidence-based practice

## Abstract

**Background:**

Given the importance of influence networks in the implementation of evidence-based practices and interventions, it is unclear whether such networks continue to operate as sources of information and advice when they are segmented and disrupted by randomization to different implementation strategy conditions. The present study examines the linkages across implementation strategy conditions of social influence networks of leaders of youth-serving systems in 12 California counties participating in a randomized controlled trial of community development teams (CDTs) to scale up use of an evidence-based practice.

**Methods:**

Semi-structured interviews were conducted with 38 directors, assistant directors, and program managers of county probation, mental health, and child welfare departments. A web-based survey collected additional quantitative data on information and advice networks of study participants. A mixed-methods approach to data analysis was used to create a sociometric data set (n = 176) to examine linkages between treatment and standard conditions.

**Results:**

Of those network members who were affiliated with a county (n = 137), only 6 (4.4%) were directly connected to a member of the opposite implementation strategy condition; 19 (13.9%) were connected by two steps or fewer to a member of the opposite implementation strategy condition; 64 (46.7%) were connected by three or fewer steps to a member of the opposite implementation strategy condition. Most of the indirect steps between individuals who were in different implementation strategy conditions were connections involving a third non-county organizational entity that had an important role in the trial in keeping the implementation strategy conditions separate. When these entities were excluded, the CDT network exhibited fewer components and significantly higher betweenness centralization than did the standard condition network.

**Conclusion:**

Although the integrity of the RCT in this instance was not compromised by study participant influence networks, RCT designs should consider how influence networks may extend beyond boundaries established by the randomization process in implementation studies.

**Trial registration:**

NCT00880126

## Background

The Randomized Controlled Trial (RCT) design has long served as the ‘gold standard’ for efficacy and effectiveness research for programs and practices designed to treat or prevent a wide array of physical and mental health problems, tested against standard conditions or other interventions. There is currently an active debate about the role that RCT designs should play in implementation and translational science. In particular, the use of randomized trials in effectiveness and implementation research has been challenged on the grounds that the ‘controlling’ part of the RCT limits important naturally occurring processes in complex systems and therefore has limited external validity [1-3]. In addition to external validity, such designs may also compromise a study’s internal validity in various ways, especially when implementors move to different organizations or transmit knowledge or skills related to implementation.

While its ultimate role is still being debated [4], there is no question that RCTs are being used in implementation research [5], particularly in comparing two different implementation strategies of an evidence-based practice (EBP) or empirically-supported intervention (ESI) [6,7]. In a recent study that relied upon an adapted randomized design, we found that the implementation of an EBP was associated with certain characteristics of the social networks of directors and senior administrators of child welfare agencies, probation departments and mental health departments [8]. Further, these networks cut across organizational and jurisdictional boundaries used to define the unit of randomization, *i.e*., the county. We concluded from this study that while the organizations may be responsible for the implementation of EBPs, the influence networks of the leaders of these organizations may represent more relevant units to consider, and possibly to randomize, in implementation research.

In the present study, we further examined these influence networks to determine whether they cut across implementation strategy conditions and thus, posed a threat to the study’s internal validity. Our objectives were to identify the network characteristics of the intervention and standard conditions of this RCT and determine the number of direct and indirect influence linkages across the two study arms.

## Methods

### Setting

The present study used data from the Cal-40 Study, a randomized implementation trial of a strategy to scale up the use of an EBP for treatment of externalizing behaviors and mental health problems (R01MH076158-01A1) [7,9]. This EBP, called Multidimensional Treatment Foster Care (MTFC) [10], has been shown to reduce out-of-home placement in group and residential care, juvenile arrests, substance abuse, youth violence, pregnancy, and behavioral and emotional problems. The implementation strategy tested was the Community Development Team (CDT) approach [11,12] to scale up MTFC in public youth serving systems in California. CDT involves peer-to-peer interactions among counties who are undergoing the training in the steps required to implement this complex program. A broker agent, the California Institute of Mental Health (CiMH), which already had relationships with all the counties, provided this CDT training. CDT was delivered to six to seven counties who worked together over a period of months to address the infrastructure, logistic and resource challenges in implementing MTFC in their counties. Comparison sites received the standard version of technical assistance for implementing MTFC without the use of CDTs; that is, each county worked alone with the MTFC purveyors who provided equivalent but individual county training. The key research question was whether the peer-to-peer CDT training would foster more rapid implementation than the standard condition. Implementation progress was measured by the Stages of Implementation Completion (SIC) [13,14].

The CAL-40 study targeted 40 California counties that had not already adopted MTFC. They were matched to form six nearly equivalent groups of six to seven counties in order to balance the design across population composition and service delivery factors. The matched groups were then randomly assigned to one of three sequential cohorts in a waitlist design with staggered start-up timelines (at months 6, 18 or 30). Further, one matched set of counties in each cohort was randomly assigned to CDT and the other to standard implementation condition, thereby generating six groups of counties with three assigned to CDT. While the trial design protocol directly inhibited CiMH from engaging in communications with standard setting counties around MTFC implementation, there were no restrictions placed on individual counties in communicating with counties in the other condition.

Embedded in the CAL-40 study was a smaller project, funded by the William T. Grant Foundation. Using both quantitative and qualitative data, we sought to accomplish the following: first, describe the structure and operation of information and advice networks of public-youth-serving systems in the first cohort of California counties; and then determine the influence of these networks in the implementation of MTFC.

### Study sample

Participants for this smaller T. T. Grant-funded study included members of the influence networks of the agencies that comprised the first cohort of counties (n = 13) of the CAL-40 Study. Agencies from counties comprising the first cohort began participating in the larger RCT in May 2007. Subsequently, directors of the child welfare, mental health, and probation departments of all 13 counties (n = 39 agencies) were invited to participate in the smaller W.T. Grant-funded study of social networks and EBP implementation. In some instances, associate directors or senior program managers were recommended by the directors to be interviewed in their place.

### Data collection

Information on the social networks of study participants was obtained from two sources, a web-based survey and semi-structured interviews. Details on the procedures for constructing networks using these two sets of data have been published elsewhere [15]. Each participant completed a semi-structured interview conducted between July and September 2008. The interview centered on knowledge and implementation of MTFC and other EBPs at the county level. Interviewees were asked if they had ever heard of the Cal-40 Project or MTFC and what their motivations were to participate or not participate in the program. Participants were then asked who they had talked to about participation in MTFC or other EBPs; prompts were given to participants as necessary to identify individuals with whom they communicated, their relationship to that person, their reasons for talking to that person, and the amount of influence that person had on their decision to participate in MTFC or a similar EBP. Then participants were asked about collaborations both within and between county agencies (*i.e*., child welfare, mental health, probation) and the nature of these collaborations. Specifically, participants were asked to identify what made for a successful versus an unsuccessful collaboration. Finally, participants were asked about who usually suggested that their agency take on new programs or initiatives. Probes to solicit influential actors included: agency staff, other agencies, community based organizations, other county officials, consultants, representatives from federal and state agencies and private foundations, treatment developers, and representatives from broker organizations including CiMH. A copy of the interview guide is found in Additional file 1. Participants’ written informed consent was obtained, and the research study was approved by the Institutional Review Board at the University of Southern California (USC UPIRB #UP-08-00033).

All those interviewed were then asked to complete a web-based social network survey to identify individuals on whom they relied for advice regarding EBP implementation. The survey asked participants to provide general demographic information (*i.e*., gender, age, number of years in occupation, current position, and time with agency). Per criteria established by Valente and colleagues [16,17], each study participant was asked to identify as many as 10 individuals for whom they have relied for advice on whether and how to use evidence-based practices for meeting the mental health needs of youth served by their agency. In order to assess whether connections across implementation strategy conditions may have been established through third-party individuals not involved with the Cal-40 study, no limitations were placed on whom could be nominated by study participants. A copy of the survey is found in Additional file 2.

### Data analysis

**Figure 1:**
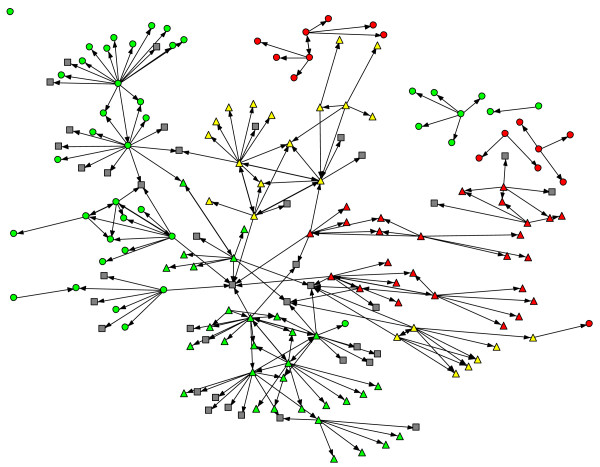
**Social network members by county treatment condition and implementation stage.** Legend: Color: green = high implementation, yellow = moderate implementation, red = low implementatinon; Shape: triangle = CDT intervention, circle = standard, square = non-county organiztion.

**Figure 2:**
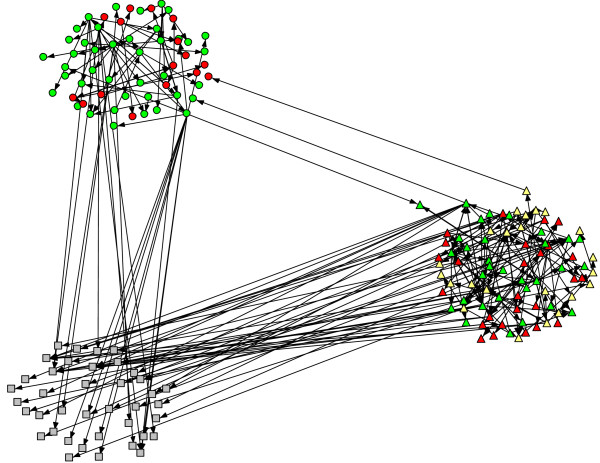
**Network by Randomization Category and Implementation Stage.** Legend: Color: green = high implementation, yellow = moderate implementation, red = low implementatinon; Shape: triangle = CDT intervention, circle = standard, square = non-county organiztion. Note: Three cross-condition paths exist between participants from 6 organizations.

The social network analysis proceeded in two stages: network visualization followed by structural analysis. A binary matrix of network ties was constructed from the two data sources. In the matrix, rows represent nominators while columns represent those nominated. A given cell is coded 0 if no tie exists and is coded 1 if person i (row) nominated person j (column) in either the web-based survey or the qualitative interview. Using this binary matrix, the network visualization was accomplished using NetDraw 2.090 [18]. The spring embedder routine was used to generate the network visualizations (presented in Figure 1). Spring embedding is based on the idea that two actors may be thought of as pushing or pulling each other; two points located close together represent actors who have a pull on each other, while distant actors push one another apart. The algorithm seeks a global optimum where there is the least stress on the ‘springs’ connecting actors to one another [19]. A second network visualization (Figure 2) was constructed to create a diagram by which individual actors are grouped by implementation strategy condition, which allowed for easy identification of connections across implementation strategy condition. This was constructed using the ‘group by attribute’ feature in NetDraw.

Network analyses were then conducted to determine whether participants in the CDT condition had more ties than those in the standard version, as hypothesized from the peer-to-peer nature of CDT. A second analysis was conducted to determine whether network ties spanned across conditions, which would be a threat to the trial's internal validity. Network analyses were conducted using Ucinet for Windows, Version 6 [20]. To understand differences between treatment and standard conditions, several network level measures of structure were assessed by treatment group, including: network size, density (the number of reported links divided by the maximum number of possible links), average distance between actors, and the number of components (*i.e*., distinct non-connected groups). To assess communication across implementation strategy conditions we opted for a set of metrics commonly used to understand communication between network actors, namely, out-degree centrality and betweenness. Out-degree centrality refers to the number of network members nominated by a particular actor. Betweenness refers to the frequency an actor falls on the shortest path connecting other actors in the network [21].

We calculated the minimum path length between any two dyads using the ‘dist’ function in the matrix algebra feature of Ucinet [20]. This creates a dyad-by-dyad assessment of minimum distance between any two nodes in the matrix. Merging this information with attribute-level information about implementation strategy condition (experimental, standard, non-county agency node), using SAS® 9.2 (Statistical Analysis Systems 9.2) [22], we then calculated the minimum distance needed for each of the agency-based actors to reach an actor of the opposite implementation strategy condition. These path lengths can be interpreted as the degree of separation [23] between two actors in different conditions, such that path length 1 represents 1 degree of separation, path length 2 represents 2 degrees of separation, and so on. Special handling of ties involving CiMH was examined as well. Because CiMH already had many relationships with county leaders and they led the training of CDT assigned counties, the trial protocol required CiMH to limit their involvement with standard implementation counties to issues not concerning MTFC implementation. Weekly meetings reinforced this limited role throughout the study, effectively closing down these potential channels of communication between CiMH and standard setting counties. We thus repeated our network analyses, removing links involving CiMH.

## Results

Of the 45 administrators from the 39 agencies in 13 counties invited to participate, 38 representing 30 agencies in 12 counties agreed to do so, yielding a response rate of 84%. The number of participants in each county ranged from two to six individuals. Some 23 (60.5%) participants were part of the CDT condition; 15 (39.5%) were part of the control condition. Table 1 presents demographic characteristics of the study participants. Participants were evenly divided between child welfare (n = 14; 36.8%), mental health (n = 12; 31.6%), and probation (n = 12 31.6%) agencies. A little over one-third were directors from their agency (n = 14; 36.8%); 8 were assistant directors (21.1%); and 16 were program managers (42.1%). The counties from which participants hailed were evenly split between small (n = 20; 52.6%) and large (n = 18; 47.4%). Approximately half were from the Bay Area (n = 18; 47.4%); 8 were from Northern California (21.1%); 10 were from Central California (26.3%); and 2 were from Southern California (5.3%). A total of 30 (86%) of those individuals who participated in semi-structured interviews also completed the web-based survey.

**Table 1:** Participant characteristics for social network data (n = 38)

**Individual characteristics**	**Control (n = 15)**	**CDT (n =23)**
Mean (SD) age in years*	50.5 (9.5)	48.7 (7.1)
Gender		
Male	6 (40.0%)	9 (39.1%)
Female	9 (60.0%)	14 (60.9%)
Agency		
Child Welfare	6 (40.0%)	8 (34.8%)
Mental Health	4 (26.7%)	8 (34.8%)
Probation	5 (33.3%)	7 (30.4%)
Position		
Director	4 (30.8%)	10 (43.5%)
Assistant Director	4 (26.7%)	4 (17.4%)
Program Manager	7 (46.7%)	9 (39.1%)
County Characteristics		
County Size		
Small	6 (40.0%)	14 (60.9%)
Large	9 (60.0%)	9 (39.1%)
Region		
Northern	5 (33.3%)	3 (13.0%)
Bay Area	7 (46.7%)	11 (47.8%)
Central	3 (20.0%)	7 (30.4%)
Southern	0 (0.0%)	2 (8.7%)
Rural County		
Yes	5 (33.3%)	10 (43.5%)
No	10 (66.7%)	13 (56.5%)
Number (SD) of participants	3.0 (0.7)	3.3 (1.6)
Network characteristics		
Proportion same county	0.810 (0.226)	
Proportion same agency	0.381 (0.266)	
Proportion same implementation stage	0.830 (0.223)	

*Information on age was missing for eight participants.

**Figure 3:**
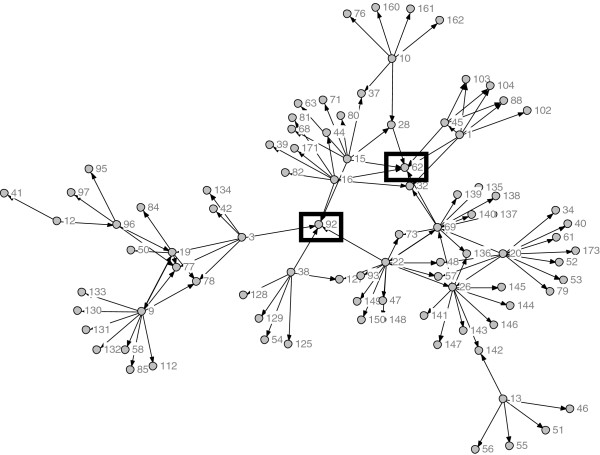
**Network of CDT Condition w/ Actors from Non-County Organizations.** Note: Nodes 62 and 92 are CiMH representatives.

**Figure 4:**
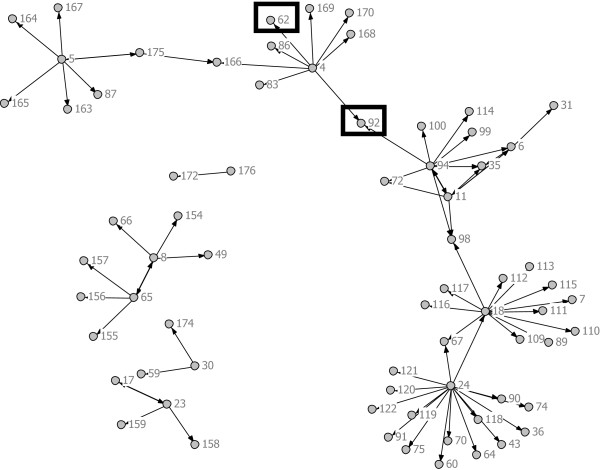
**Standard Condition w/ Actors from Non-County Organizations.** Note: Nodes 62 and 92 are CiMH representatives.

The total network, depicted in Figure 1, contained 176 individuals. A total of 45 percent (n = 80) were in the CDT group, 33% (n = 57) were in the standard group, and 22% (n = 39) could not be classified since they were affiliated with a non-county organization (*e.g*., CiMH). Figures 3 and 4 depict the CDT and standard condition networks (including those non-county actors who may have been nominated). It is evident by visual comparison of these two networks that the CDT network is more interconnected than the network involving the standard group, which is fragmented into several disconnected components. This is in keeping with the peer-to-peer approach taken by CDT. Table 2 presents the social network metrics of the CDT and standard conditions. Although the CDT network (105 nodes) and the standard network (98 nodes) were roughly the same size, the CDT network had more links than the standard network (114 vs. 77). The density of the CDT network was also slightly higher than the standard network (0.0104 vs. 0.008). Not including isolates, the CDT condition had fewer components (1) compared to the standard group (5), indicating a more cohesive structure. Likewise, raw out-degree centrality was higher in the CDT group, indicating greater communication among actors in that condition relative to the standard condition. Average distance between any two nodes was slightly higher among the implementation strategy condition (1.748) than the standard condition (1.368), and betweenness was higher among the CDT group. These latter two results reflect the greater number of discrete components in the standard condition. Since there was only one component in the CDT group, most nodes eventually connect. Both betweenness and average node distance are greater in that network, reflecting this ‘larger reach’ of the CDT network. When the two representatives of CiMH were excluded, the number of components in the CDT condition increased from one to two, indicating the importance of CiMH in forming bridges, and the number of links decreased to 103; however, the other network metrics were essentially unchanged.

**Table 2:** Comparison of treatment and standard conditions (w/ non-county actors) with and without CiMH representatives

	**With CiMH nodes**		**Without CiMH nodes**	
Metric	Control	CDT	Standard	CDT
Size	98	105	96	103
# of steps	77	114	73	103
# of components^1^	5	1	6	2
Density	0.008	0.0104	0.0081	0.0098
Average distance^2^	1.368	1.748	1.358	1.769
Out-degree centrality	0.786 (2.508)	1.086 (2.750)	0.771 (2.447)	1.000 (2.558)
Betweenness centrality	0.429 (1.654)	1.667 (7.045)	0.406 (1.545)	1.612 (6.719)

^1^Isolates not counted as components.

^2^Among reachable pairs.

Post-hoc analyses using pairwise independent sample t-tests showed no statistically significant differences in out-degree centrality between the two networks (CDT mean = 1.899, SD = 3.510 versus Standard mean = 1.259, SD = 3.160). However, average betweenness centrality was greater in the CDT condition (mean = 5.544, SD = 14.883) compared to the standard condition (mean = 0.741, SD = 2.970) (t = 2.37; p = 0.0191). This statistically significant result (p <0.01) demonstrates the high interconnectivity of the peer-to-peer networked CDT group compared to the standard group. Statistically significant differences between the two conditions on betweenness centrality remained even after CiMH actors were removed from the network.

Table 3 presents the distribution of individuals with respect to the minimum path length required to reach another individual in the other arm of the study. These minimum path lengths could (and often did) contain paths, which included one of the 39 non-randomized nodes. Restricting the analysis to the 137 individuals from agencies participating in the RCT who were assigned to either treatment or standard condition, only 6 (4.4%) individuals were directly connected to an individual (and by extension, their agency) in the opposite implementation strategy condition. Most individuals, however, had indirect steps to individuals in the opposite condition. In particular, 104 (75.9%) of the individuals were connected to an individual in the opposite condition in less than four degrees of separation. However, 30 (21.9%) individuals never reached an individual in the opposite condition, even via indirect connections. When links with the two CiMH representatives are excluded, the percentage of individuals connected to an individual in the opposite condition in less than four degrees of separation decreases to 59.9 percent (n = 80), and the percentage of individuals never reaching an individual in the opposite condition increases to 25.6 percent.

**Table 3:** Minimum path length connecting organizations in opposing implementation strategy conditions, with and without CIMH nodes (n = 137)

**Minimum path length**	**With CiMH nodes**		**Without CiMH nodes**	
	N	%	N	%
1	6	4.38	6	4.38
2	19	13.87	14	10.22
3	64	46.72	46	33.58
4	15	10.95	16	11.68
5	3	2.19	17	12.41
6	0	0.00	3	2.19
Never Connect	30	21.9	35	25.55

It is useful to look at the three dyads that cross intervention boundaries as illustrated in Figure 2 where the shape of the node indicates condition (triangle for CDT, circle for standard, and square for other organizations), and the color represents implementation stage (low = red, moderate = yellow, high = green). One leader in a CDT county sought advice about EBPs from another leader in a standard county; both counties were at the highest stage of implementation as of October 2010. Another leader in a CDT county at a moderate level of implementation sought advice from a leader in a standard county at a lower stage of implementation. A third leader in a standard county at a high level of implementation sought advice from a CDT county leader at the same stage of implementation. Two of the other pairs came from similar regions (Northern and Southern California). Two participants from one CDT county were directly connected to individuals from standard counties. Figure 2 also shows the higher density of steps among leaders in the CDT condition compared to that for the standard condition. There are a substantial number of steps that CDT leaders have with others who are not part of the formal study, even when we exclude the CiMH brokers who have a special role in this project.

When individuals in counties not participating in the RCT are excluded from analysis, the networks in the CDT condition and the standard condition take the forms as illustrated in Figures 3 and 4, respectively. Removal of Node 92 leads to the development of two additional components (unconnected networks) in the CDT condition and a complete separation of the largest component in the standard condition into two components.

Finally, we conducted a post hoc analysis of these data in which we limited the network to the original 38 study participants. One participant had a direct tie to a participant in a different implementation strategy condition, and a second participant had a two link path to a participant in a different condition. The remaining 34 participants never connected to a participant in the opposing implementation strategy condition. Limiting the analyses to these 38 nodes ignores the potential for actors between conditions to be linked indirectly.

## Discussion

Interpersonal contacts within and between individuals in organizations and communities are important influences on the adoption of new behaviors [24-26]. Based on Diffusion of Innovations Theory [22] and Social Learning Theory [27], Valente’s [28] social network thresholds model calls for identification of champions within peer networks that manage organizational agenda setting, change, and evaluation of change (*e.g*., data collection, evaluation, and feedback). Studies and meta-analyses have also shown that both the influence of trusted others in one’s personal network and having access and exposure to external information are important influences on rates of adoption of innovative practices [29-32].

This study found relatively few direct crossover contacts between system leaders in the two study conditions, suggesting that the integrity of the RCT was not compromised by the existence of overlapping influence networks. Only six individuals had direct (first order) steps with individuals in the other condition, and the relationships among these six individuals appeared to be unrelated to the stage of implementation of MTFC in their respective counties. However, there are many more indirect connections between the two study conditions indicating potential threats to the internal validity of the study due to communication between and possible influence of system leaders on each other through third-party actors. In fact, system leaders of child public service systems have much in common in terms of the complex and multifaceted issues and problems that they routinely deal with, and it is not surprising that they go to each other for advice and support. Therefore, in this randomized trial as in others, the potential for contamination through cross condition network ties is a clear concern when grouping of contrasting conditions occurs within contexts that are in close social proximity of each other (*e.g*., in classrooms within the same schools, therapists in the same mental health clinics, professional relationships in social service settings). Such threats to internal consistency are real and should be considered and measured as part of these trials.

There were very few direct ties in this study across condition and only a modest number of second degree ties. Most of the potential for crossover effects in the present study would have happened through third-party actors, who were not affiliated with a specific county. For example, one of the most popular connections between the two implementation strategy conditions was an actor within CiMH. This finding is not surprising as one of the roles of CiMH is to provide information to all counties regarding best practices. These results indicate that when conducting RCTs in which the county is the unit of randomization, specific instructions to non-county agency leaders that specify limiting communication of treatment practices across conditions will improve the integrity of the study design. However, this recommendation should be interpreted with caution given the real-world requirements of these third-party agencies. If a particular practice is working in an implementation strategy condition, it may be unethical to withhold information regarding this practice from non-treatment counties simply to adhere to randomization procedures of research. In this particular implementation trial, the issue of limiting information through CiMH communications did not raise ethical issues because the remaining counties were provided with the standard implementation model and the same evidence-based intervention.

In the current study, there is no evidence to suggest that the observed crossover had direct effects on the study results. In fact, if the results from the main study indicate that there are differences in outcomes from the CDT and standard implementation conditions, the concerns about internal validity threats will be obviated. However, the results of this study also show that the randomized trial protocol disrupted some of the naturally occurring influence networks in the standard condition, at least with regard to CiMH. Also, most of the study participants and the organizations they represent had indirect steps to organizations in the opposite condition. In particular, 104 (75.9%) of the participants were connected to a participant and organization in the opposite condition in less than four degrees of separation. These connections occurred through individuals or organizations not part of the study. As these non-study network members serve as important sources of information and advice [33], and as this information and advice appears to be associated with implementation outcomes [8], the exclusion of this segment of the social networks of either implementation strategy condition creates the potential for attenuating the effect of the implementation strategy by limiting existing network influences.

In the context of this particular study, the role of this potential confounder is illustrated by the results from the post hoc analyses showing significant differences in the network segments of individuals and organizations that were randomized into the two implementation strategy conditions. The network of the counties in the CDT condition had fewer components (one versus five), exhibited greater size and density, and significantly greater betweenness centrality than the network in the standard condition. These results confirm, as we had hypothesized, that there was more cohesion and connectivity among members of the CDT condition than among members in the standard condition. This result is consistent with one of the six core processes of the CDT model, which is the use of Peer-to-Peer exchange and support to promote engagement, commitment, and learning by a group of sites, and encourage cross-fertilization of ideas [11]. As the CDT counties had participated in two CDT meetings prior to data collection, these results suggest that the intervention was successful in creating or strengthening network steps among counties engaged in the shared goal of implementing MTFC. Furthermore, network size and centrality were found to be significant independent predictors of implementation stage in an earlier study of this cohort [8]. Inasmuch as one of the aims of the CDT intervention is to foster the development of influence networks for the purpose of facilitating EBP implementation, and that such networks are associated with the pace of implementation, it becomes particularly important to take into consideration in both design and analysis the roles of influence networks that are not directly involved in the RCT but which nevertheless may influence the study outcomes.

There are several limitations to our study that deserve mention. First, this investigation was conducted during the initial stage of EBP implementation with a small number of counties. Although our findings suggest that there will be changes in patterns and processes of implementation over time, we were primarily interested in examining networks at the initial stages of the implementation process. Second, systems leaders who participated in interviews at this stage of the CAL-40 Study represent almost all of the first cohort, and while this cohort was created to match the population of systems leaders participating in other cohorts, they may not represent the broader population of systems leaders engaged in child and adolescent mental health services across the country. Thus, the results obtained thus far may not generalize to systems outside of California. Third, the 176-member network was constructed based on information from 38 interviewees who were not asked to provide information on sociodemographic and occupational characteristics on those they nominated. In this context, we do not need responses from all 176 members to create the network of linkages between organizations. Our networks, however, are limited in their capacity to show bi-directionality of ties, as most nodes were not interviewed and so linkages are only established by the 38 interviewees. There may be a large number of linkages among the nominated-and-not-interviewed nodes which are missed. Inclusion of these missing linkages, however, would only serve to shorten the number of steps between particular organizations; hence our network is a conservative one, with respect to diffusion across conditions. Consequently, we lacked individual-level measures on the nodes who were not directly interviewed, thereby limiting our statistical power to examine the influence of such characteristics as predictors of network structure. Further, although it was beyond the scope of this study, understanding of the nature and content of relations between the individuals belonging to these networks might have been facilitated by the qualitative information obtained from the semi-structured interviews. Use of qualitative information in this manner in future research on the role of influence networks in evidence-based practice implementation is recommended. Finally, network size and structure may have been influenced by factors such as the number of participants in each county and leadership role (Director, Associate Director, Program Manager). Although we found no difference in the presence of such factors across experimental condition, future studies utilizing RCT designs may wish to examine their potential influence in understanding group differences in social network size and structure.

## Conclusions

Despite these limitations, the results of this study suggest that the integrity of the RCT in this instance was not compromised by influence networks of study participants. Nevertheless, RCT designs should take into account the fact that influence networks that are associated with implementation outcomes extend beyond boundaries established by randomization process.

## Competing interests

PC is a partner in Treatment Foster Care Consultants, Inc., a company that provides consultation to systems and agencies wishing to implement MTFC.

## Authors’ contributions

LAP is the principal investigator of the Social Network Study. He collected the qualitative data, supervised the analysis of the qualitative data and collection and analysis of the survey data, and contributed substantially to the writing of the manuscript. IWH, ER, and CHB contributed substantially to data analysis and the writing of the manuscript. PC is the principal investigator of the parent study randomized trial that forms the basis for this study and contributed to the conceptualization, design and writing of the manuscript. All authors read and approved the final manuscript.

## Supplementary Material

Additional file 1 Semi-Structured Interview Guide for Agency Directors. Click here for file

Additional file 2 Social Network and EBP Implementation Web-Based Survey. Click here for file
