# Supplementary material for: Influence network linkages across implementation strategy conditions in a randomized controlled trial of two strategies for scaling up evidence-based practices in public youth-serving systems
Source: Implement Sci. 2013 Nov 14;8:133. doi: 10.1186/1748-5908-8-133 (PMC3930152; doi:10.1186/1748-5908-8-133)
Supplement: Additional file 1 — Semi-Structured Interview Guide for Agency Directors. [file 1748-5908-8-133-S1.docx]

**Additional File 1: Semi-Structured Interview Guide for Agency Directors**

**Introduction:** Thank you once again for agreeing to participate in this project. I do not want to waste your valuable time, so I have prepared some questions that I would like to ask you over the next hour or so. You can spend as much or as little time as you like answering each question. It is less important that you answer every question than it is to tell me all you can about the issues raised by any question you feel to be important and relevant to your experience with the CAL-40 Project and the decision to participate or not participate.

1. Have you ever heard of the CAL-40 Project or MTFC before you were contacted about this study? (IF NOT SKIP TO QUESTION 6)

- How did you first hear about the CAL-40 Project?
- Who contacted you about participating?
- Do you remember when they contacted you?
- Do you recall what was said about the project at the time you were first contacted?

1. What factors motivated you to participate/not participate in the CAL-40 Project?

- Did your decision have anything to do with how you were invited to participate?
- Did your decision have anything to do with who invited you to participate?
- Did your decision have anything to do with what you know or have heard about MTFC?
- Did your decision have anything to do with your previous experience with adopting evidence-based practices like MTFC?
- Did your decision have anything to do with the needs of your clients?
- Did your decision have anything to do with the needs or desires of your staff?
- Did your decision have anything to do with your agency’s ability to afford to use MTFC?
- Were there any other reasons why you decided to participate/not participate?

1. Can you think of anyone beside the investigators or representatives from CIMH who you talked to about participating in the CAL-40 Project?

- Who did you talk to?
- What relation is this person/are these persons to you? (e.g., friend, co-worker, superior, subordinate)?
- Why did you talk to that/those people?
- What did they tell you?
- Did what they told you have any influence on your decision to participate?
- How important is what they told you relative to the other reasons for participating/not participating you listed earlier?

1. For participants only

- Since agreeing to participate, have you encountered any barriers to participating?
- What are they?
- Have you been able to overcome these barriers? How?
- Has your staff been motivated to participate in this project?
- Have you done anything to motivate your staff to participate?

1. Is there anything else about MTFC you think it would be important to know before deciding whether or not to use it?
2. Has your agency implemented any new programs or taken on any new initiatives in the past year?

- Could you please describe them?
- Did you solicit any information and advice from anyone before beginning this project?
- Who did you talk to? (within county, other counties)

1. Did any of these new initiatives involve collaborations with the other agencies (Child Welfare, Mental Health, Probation)?

- Are you currently running any new programs with these other agencies?
- How would you characterize the collaborations you have with these other agencies?
- What would you say are the most important elements of a successful collaboration?
- Have you ever been part of a collaboration that did not go so well? Could you tell me about them?

1. Have any of the following ever suggested to you to take on new programs or initiatives?

- Your own staff
- Other agencies
- The CBOs in your county
- Local judges
- Consumer advocates

1. Do representatives from other counties ever contact you for advice and information about programs they are thinking of starting? Which ones?
2. Finally, what do you think is necessary for this county to successfully get programs for kids up and running?
3. Please provide us with your email address so that we can invite you to participate in a brief web-based survey on what people you rely upon for advice in using evidence-based practices like MTFC.

Thank you once again for your time.
