# Supplementary material for: Influence network linkages across implementation strategy conditions in a randomized controlled trial of two strategies for scaling up evidence-based practices in public youth-serving systems
Source: Implement Sci. 2013 Nov 14;8:133. doi: 10.1186/1748-5908-8-133 (PMC3930152; doi:10.1186/1748-5908-8-133)
Supplement: Additional file 2 — Social Network and EBP Implementation Web-Based Survey. [file 1748-5908-8-133-S2.doc]

**Additional File 2: Social Network and EBP Implementation Web-Based Survey**

Thank you for agreeing to participate in this survey. As we explained in our previous communication, it should take you approximately 10-15 minutes to complete this survey. Please answer the following:

Your name: ______________________________________

Your age: ________ years

Your gender: ___ male ____ female

Your occupation: _________________________________

Your employer: __________________________________

Location of your employer: __________________ County

Your position: ___________________________________

Number of years employed in this position: ____________

Number of years employed in this agency: ____________

Number of years employed in this occupation: __________

Do you have any role in or responsibility for implementing Multidimensional Treatment Foster Care (MTFC) in your County?

Yes ____ No ___

Have you or your staff ever used MTFC as a practice? Yes ____ No ____

Instructions: Please provide as much information as possible when filling in the boxes below: In column 1, name as many as 10 individuals for whom you have relied for advice on whether and how to use evidence-based practices for meeting the mental health needs of youth served by your agency. If you have relied on more than 10 individuals, just name the first 10 that come to mind. If you participated in the interview portion of the study, please provide the email addresses of these individuals so that we may also invite them to participate. If you were not interviewed, you do not have to provide any email addresses for the individuals you list.

In the remaining columns, simply click (check) on the following if they apply to the name you have listed in column 1.

1. Which of these individuals have you sought advice from with respect to MTFC or the CAL-40 Study?
2. Which of these individuals works for the same agency as you?
3. Which of these individuals works for another agency or employer?
4. Which of these individuals do you regard as a subordinate or employee of yours?
5. Which of these individuals do you regard as your superior or supervisor?
6. Which of these individuals do you regard as a friend?

|  | a | b | c | d | e | F |
| --- | --- | --- | --- | --- | --- | --- |
| Name and email address* | Sought advice from with respect to MTFC or CAL-40 | Works for same agency as you | Works for another agency or employer | Is a subordinate or employees of yours | Is your superior or supervisor | Is your friend |
|  |  |  |  |  |  |  |
|  |  |  |  |  |  |  |
|  |  |  |  |  |  |  |
|  |  |  |  |  |  |  |
|  |  |  |  |  |  |  |
|  |  |  |  |  |  |  |
|  |  |  |  |  |  |  |
|  |  |  |  |  |  |  |
|  |  |  |  |  |  |  |
|  |  |  |  |  |  |  |

* Please provide email address only if you participated in the interview part of this study.
